# The complete chloroplast genome of a fast-growing tree *Lophostemon confertus* (Myrtaceae)

**DOI:** 10.1080/23802359.2022.2158691

**Published:** 2023-01-02

**Authors:** Wenhang Su, Rifan Liang

**Affiliations:** Department of Art and Design, Guangxi Vocational & Technical College, Nanning, Guangxi, China

**Keywords:** *Lophostemon confertus*, ornamental tree, Myrtaceae, chloroplast genome, phylogenetic analysis

## Abstract

*Lophostemon confertus* (Myrtaceae), a fast-growing ornamental tree, is widely cultivated in tropical and subtropical regions. To determine its phylogenetic position within Myrtaceae, here we report its complete chloroplast (cp) genome, which is 160,297 bp long and contains two inverted repeats (IRs) of 26,490 bp each, separated by a small single-copy region of 18,826 bp and a large single-copy region of 88,491 bp. The cp genome contains 123 genes, including 73 unique protein-coding genes (six duplicated in the IR regions), 29 unique tRNA genes (seven duplicated in the IR regions), and four unique rRNA genes (all located in the IR regions). Phylogenetic analysis of 18 species of Myrtaceae showed that *L. confertus* is sister to *Xanthostemon chrysanthus.* The complete cp genome of *L. confertus* provides a valuable genetic resource for further phylogenetic studies.

## 1. Introduction

*Lophostemon confertus* (Br.) Peter G. Wilson & J. T. Waterhouse (1982), a fast-growing and popular ornamental tree, is native to Australia, and is widely cultivated in tropical and subtropical regions. Due to its rapid growth, evergreen leaves, and air pollution resistance, it is used as a multipurpose tree, from an ideal landscape resource in scenic zones and city roads to excellent pioneer species for wasteland afforestation. Moreover, the superior quality of its timber, like hard, moisture-proof and termite-resistant, endows it with important economic value, and becomes an important economic tree in China. Sufficient genome resources can not only do great help to species identification and phylogenetic analysis, but also provide a molecular basis for marker-assisted breeding and horticultural variety improvement. However, little genetic and genomic information is available for this species, and more generally, even neither for the tribe Leptospermoneae to which it belongs. In this study, the complete chloroplast of *L. confertus* was first assembled through genome skimming sequencing data and its phylogenetic position within Myrtaceae was resolved.

## 2. Materials and methods

**Figure 1.**
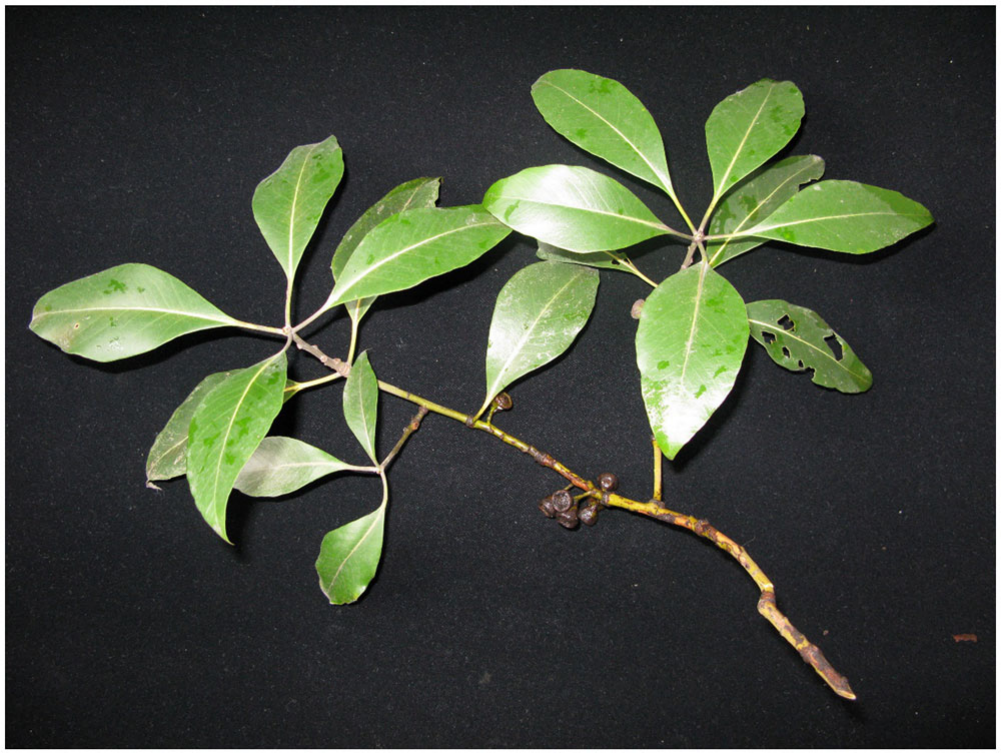
A branch of *Lophostemon confertus* showing the morphology of leaves and fruits. Photographed by Wenhang Su.

**Figure 2.**
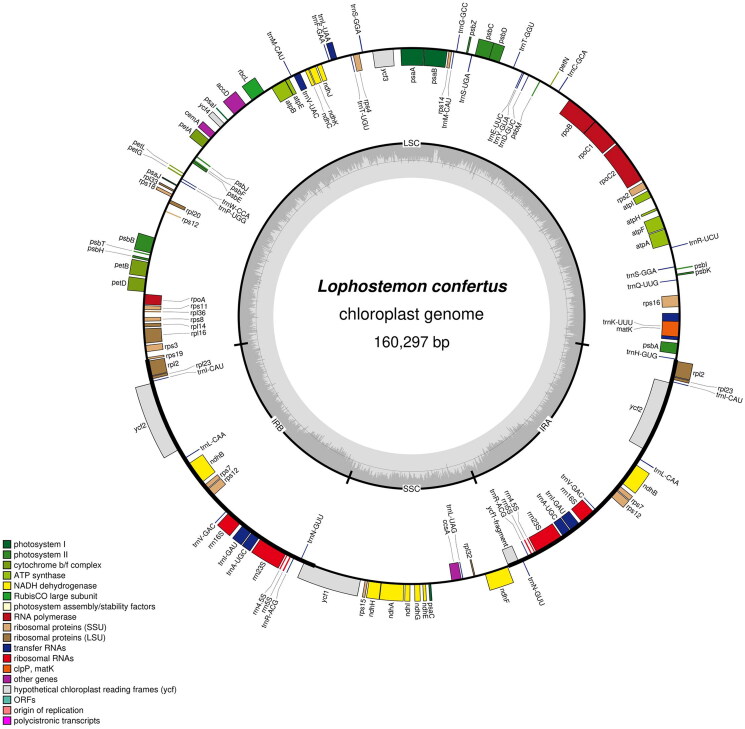
The chloroplast genome map of *Lophostemon confertus*. Genes are shown outside and inside the outer circle are transcribed counterclockwise and clockwise, respectively. GC and AT contents across the chloroplast genome are shown with dark and light shading, respectively, inside the inner circle.

**Figure 3.**
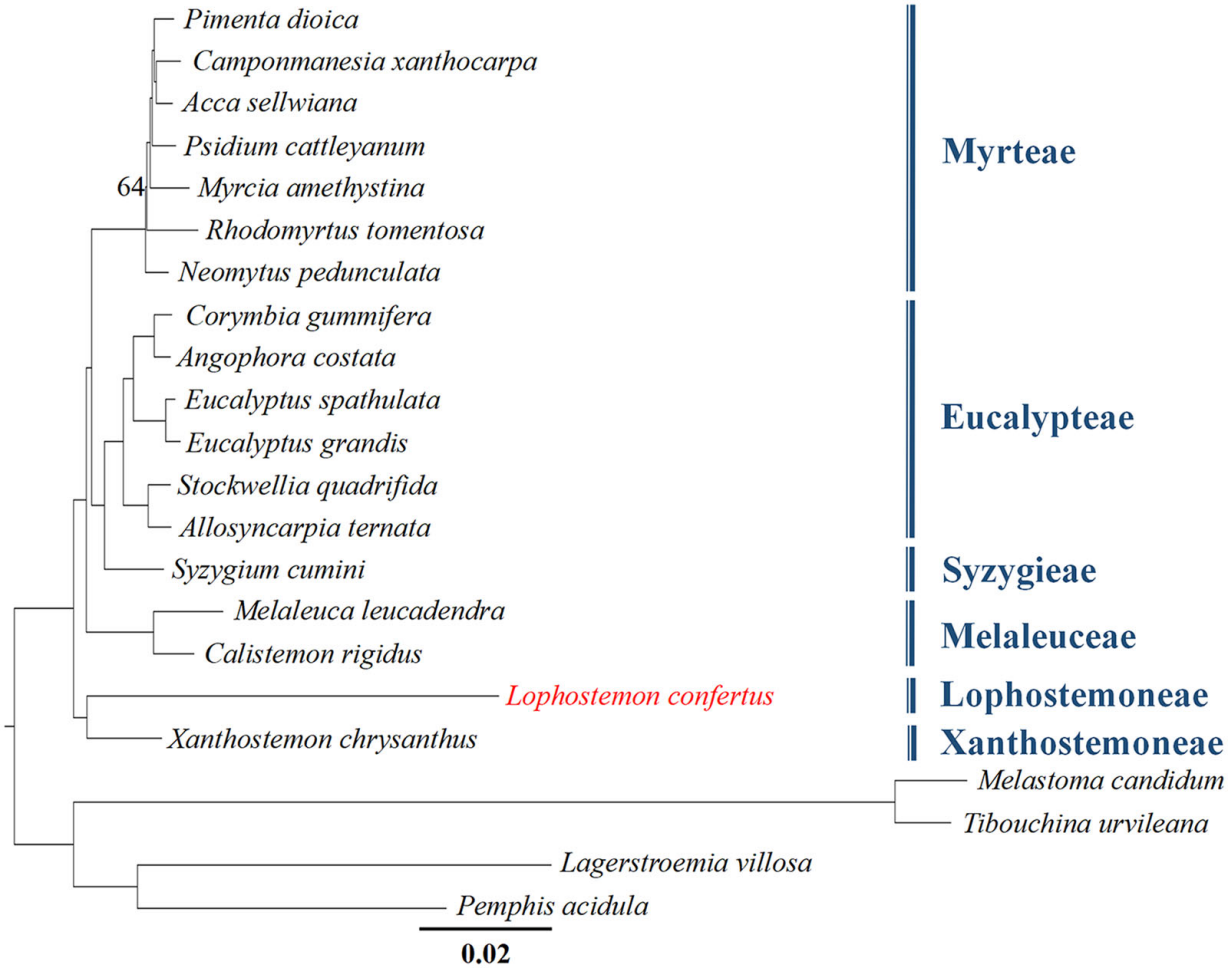
Phylogenetic tree based on the complete chloroplast genome sequences of 18 species from Myrtaceae, with four species from Melastomateae and Lythraceae, used as outgroups. Bootstrap support values of all but one node are 100 (omitted here), and only one node with a bootstrap support value of 64 is shown. The following sequences were used: *Pimenta dioica* KY085891.1 (unpublished), *Camponmanesia xanthocarpa* KY392760.1 (unpublished), *Acca sellwiana* KX289887.1 (Machado et al. 2017), *Psidium cattleyanum* MN095413.1 (Rodrigues et al. 2020), *Myrcia amethystina* MW353255.1 (Lima et al. 2021), *Rhodomyrtus tomentosa* MK044696.1 (Huang et al. 2019), *Neomytus pedunculata* MW214671.1 (Maurin et al. 2022), *Corymbia gummifera* KC180800.1 (Bayly et al. 2013), *Angophora costata* KC180805.1 (Bayly et al. 2013), *Eucalyptus spathulata* KC180793.1 (Bayly et al. 2013), *Eucalyptus grandis* MT700491.1 (unpublished), *Stockwellia quadrifida* KC180807.1 (Bayly et al. 2013), *Allosyncarpia ternata* KC180806.1 (Bayly et al. 2013), *Syzygium cumini* GQ870669.3 (unpublished), *Melaleuca leucadendra* MT700493.1 (unpublished), *Calistemon rigidus* MN794317.1 (Liu et al. 2020), *Xanthostemon chrysanthus* MW837774.1 (unpublished), *Melastoma candidum* KY745894.1 (Ng et al. 2017), *Tibouchina urvileana* NC043810.1 (Gonçalves et al. 2019), *Lagerstroemia villosa* MK881633.1 (Gu et al. 2019), *Pemphis acidula* MH727532.1 (Jian and Ren 2019).

The leaves and fruits of *Lophostemon confertus* were photographed by ourselves and shown in Figure 1. This species is not an endangered or protected species, so no permissions are required to obtain the sample. Fresh young leaves of an individual *L. confertus* were collected from the campus of Sun Yat-sen University (N113°18'8″, E23°5'24″), Guangzhou, China. A voucher specimen was identified by Rifan Liang and deposited at Sun Yat-sen University Herbarium (SYS) (http://cfh.ac.cn/subsite/default.aspx?siteid=SYS) with the specimen voucher code SYS-Bore-2021-10–13 under the charge of Wenbo Liao (lsslwb@mail.sysu.edu.cn). Total DNA was extracted from fresh leaves using the CTAB method (Doyle and Doyle 1987). Genomic DNA was fragmented into ∼350 bp fragments by an ultrasonic processor to construct a DNA library, which was then sequenced on an Illumina NovaSeq 6000 platform. Approximately 5.0 Gbp of paired-end sequences (2 × 150 bp) were generated and filtered using Trimmomatic (Bolger et al. 2014). Clean reads were used to assemble the chloroplast genome using GetOrganelle v1.6.4 with default parameters (Jin et al. 2020) by de novo algorithm without a reference genome. To clarify the accuracy of the assembly, we further mapped our clean reads back to the assembled chloroplast (cp hereafter) genome to assess the depth of coverage (Figure S1). Culminated in stitching into a synthetic loop, the chloroplast genome was automatically annotated by a web server CPGAVAS2 (Shi et al. 2019) and double-checked by Geneious version 11.1.5 (Kearse et al. 2012) followed by manual adjustment and confirmation. The structures of intron-containing genes were visualized in CPGVIEW (http://www.1kmpg.cn/cpgview, unpublished). OGDRAW (Lohse et al. 2013) was applied to visualize the gene map of *L. confertus* (Figure 2). To infer the phylogenetic position of *L. confertus* in Myrtaceae, the complete cp genome sequences of 18 species in this family (including *L. confertus*) were used for phylogenetic reconstruction. Two species each from Melastomataceae and Lythraceae were used as the outgroups. All the complete cp genome sequences of these species except *L. confertus* were downloaded from GenBank with their accession numbers shown in Figure 3. After sequence alignment with MAFFT v7.307 (Katoh and Standley 2013), a maximum likelihood (ML) tree was constructed based on the GTRGAMMA substitution model with 1000 bootstrap replicates using RAxML v8.2.12 (Stamatakis 2014).

## 3. Results

The complete cp genome of *L. confertus* (GenBank accession No. OM640421.1) was 160,297 bp long, with an overall GC content of 36.81%. It contained two inverted repeats (IRs) of 26,490 bp each, separated by an 18,826 bp small single-copy (SSC) region and an 88,491 bp large single-copy (LSC) region. It has 123 genes, including 73 unique protein-coding genes (six duplicated in the IR regions), 29 unique tRNA genes (seven duplicated in the IR regions) and four unique rRNA genes (all located in the IR regions), in which *rps12* and *ycf3* had two introns (Figure S2). Like other angiosperms, *L. confertus* exhibits trans-splicing in *rps12*.

As shown in the phylogenetic tree (Figure 3), the relationships of the six tribes of Myrtaceae with available cp genome data are well resolved, and *L. confertus* aligns as a sister group to *Xanthostemon chrysanthus*, both with high bootstrap support.

## 4. Discussion and conclusion

The cp genome of *Lophostemon confertus* (Myrtaceae), a fast-growing roadside tree, was first sequenced and assembled in this study. Phylogenetic analysis of 18 species of Myrtaceae showed that *L. confertus*, which belongs to the tribe Leptospermoneae, is sister to *Xanthostemon chrysanthus* from another tribe Xanthostemoneae.

Myrtaceae, consisting of 17 tribes and about 5650 species, is one of the largest families of flowering plants (Wilson et al. 2005; Govaerts et al. 2015). The phylogeny of this family has attracted much attention, but most of the studies were conducted based on the use of very few molecular markers, such as nuclear ribosomal internal transcribed spacers (nrITS) (Lucas et al. 2007; Biffin et al. 2010), *matK* (Wilson et al. 2005) and *ycf2* (Machado et al. 2020). Recent phylogenetic analyses based on complete cp genome sequences have been performed mainly from the species-rich tribes Myrteae, Eucalypteae and Syzygieae (Bayly et al. 2013; Machado et al. 2017; Machado et al. 2020; Balbinott et al. 2022). To date, no species from the tribe Lophostemoneae has available cp genome sequences. The complete cp genome of *L. confertus* provides a valuable genetic resource for comprehensively resolving the phylogenetic relationships among 17 tribes of Myrtaceae in near future.

## Ethical approval

No ethical issues were involved in this study. The collection of plant sample was legal and reasonable. A voucher specimen has been identified by Rifan Liang and deposited at Sun Yat-sen University Herbarium (SYS) with the specimen voucher code SYS-Bore-2021-10-13 under the charge of Wenbo Liao (lsslwb@mail.sysu.edu.cn). Information on the voucher specimen and who identified it were included in the manuscript.

## Supplementary Material

Supplemental Material

Supplemental Material

## Data Availability

The data that support the findings of this study are openly available in NCBI at https://www.ncbi.nlm.nih.gov/OM640421 under the accession no. OM640421. And the associated Bioproject, SRA, Bio-sample numbers are PRJNA804362, SRR17931549 and SAMN25731117, respectively
