# Supplementary figures and images for: The complete chloroplast genome of a fast-growing tree Lophostemon confertus (Myrtaceae)
Source: Mitochondrial DNA B Resour. 2023 Jan 2;8(1):26–9. doi: 10.1080/23802359.2022.2158691 (PMC9815246; doi:10.1080/23802359.2022.2158691)

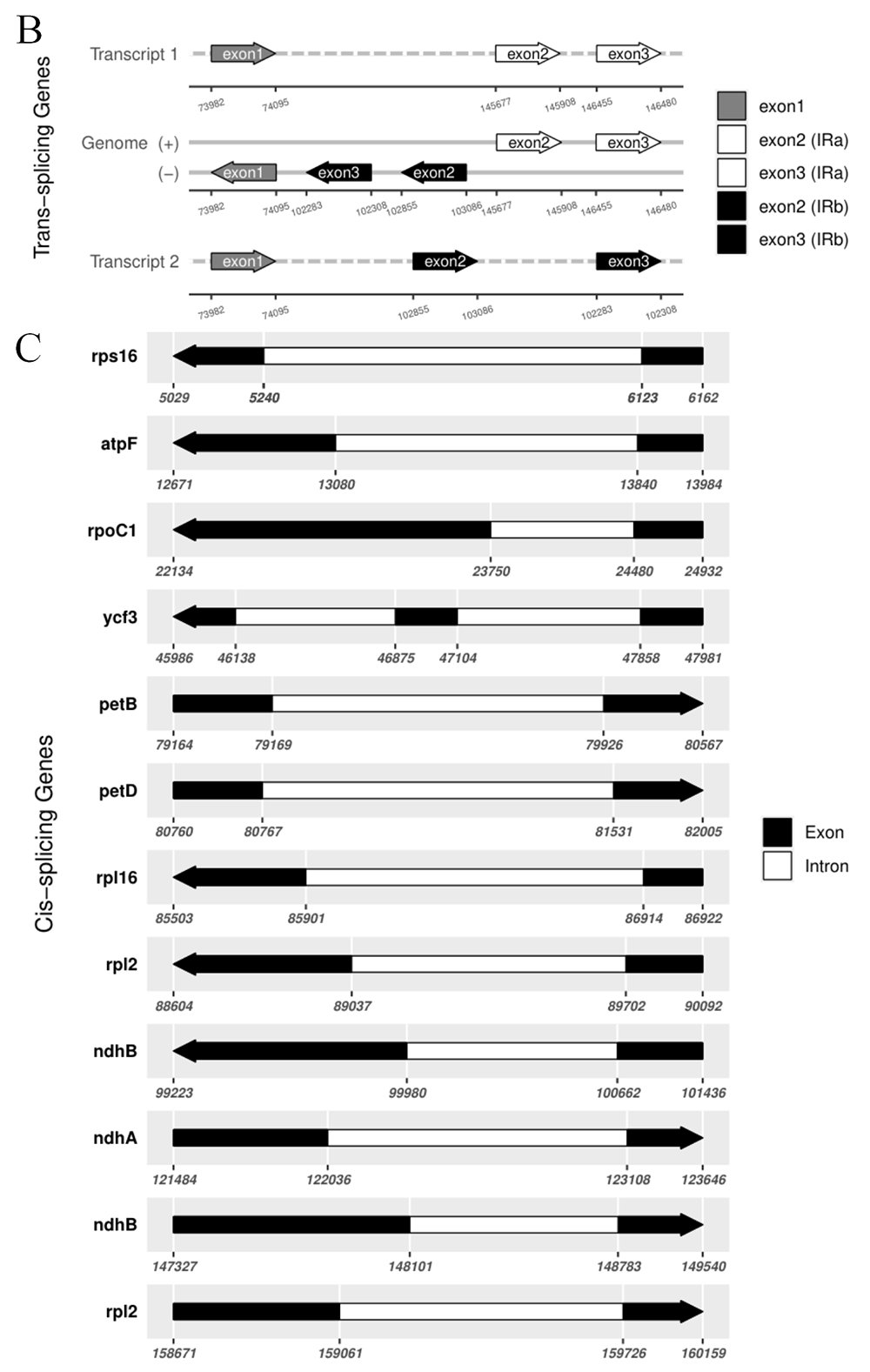

Supplement: Supplemental Material [file TMDN_A_2158691_SM4627.png]

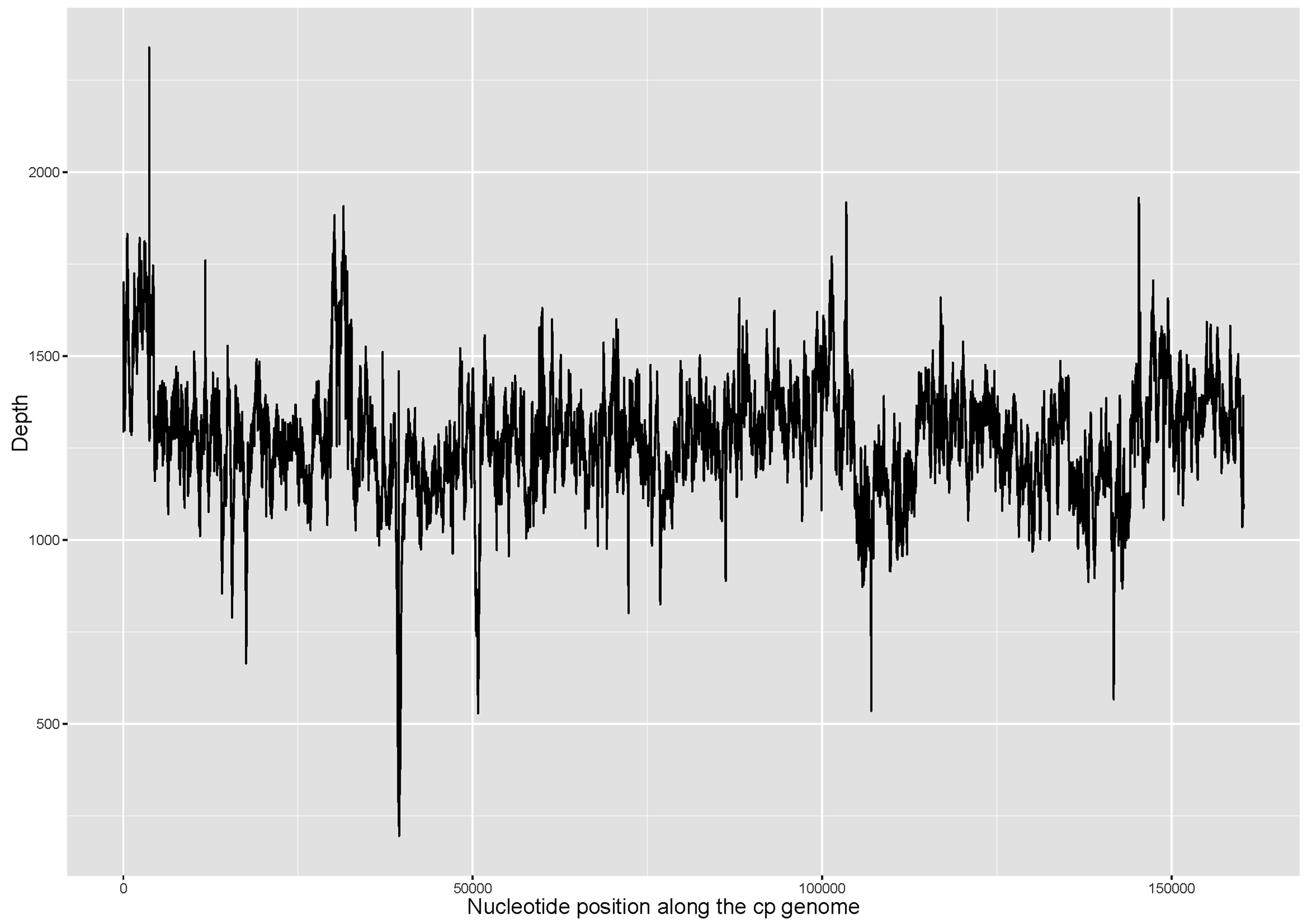

Supplement: Supplemental Material [file TMDN_A_2158691_SM4626.png]
